# Combined Arthroscopic Anterior Cruciate Ligament and Oblique Popliteal Ligament Reconstruction of the Knee

**DOI:** 10.1002/atn2.70346

**Published:** 2026-11-10

**Authors:** Farzad Amouzadeh Omrani, Mohammad Mahdi Sarzaeem, Alireza Manafi Rasi, Mohammad Mahdi Omidian, Ali Alahgholiei, Mohammadreza Dolikhani, Ali Pourmojarab

**Affiliations:** ^1^ Department of Orthopaedic Surgery and Traumatology School of Medicine, Shahid Beheshti University of Medical Sciences Tehran Iran

## Abstract

The oblique popliteal ligament is the largest ligamentous structure of the posterior knee capsule and serves as a primary restraint to genu recurvatum and a contributor to rotatory stability. This technical note describes an arthroscopic‐assisted method for oblique popliteal ligament reconstruction, performed as an isolated procedure or in combination with anterior cruciate ligament, posterior cruciate ligament, or multiligament knee reconstructions. Indications include symptomatic post‐traumatic genu recurvatum, posterior capsular laxity, recurrent or high‐risk anterior cruciate ligament failure associated with hyperextension, persistent posterior or rotational instability, and complex multiligament injuries. The technique uses standardized femoral and tibial tunnels; controlled posterior graft passage through posteromedial, trans‐septal, and posterolateral portals; and fixation in slight knee flexion to optimize posterior capsular tension in full extension. The procedure is designed to restore native posterior knee biomechanics while minimizing neurovascular risk. Oblique popliteal ligament reconstruction may represent a valuable adjunct in managing selected cases of knee instability characterized by hyperextension and posterior capsular deficiency.

## Graphical abstract

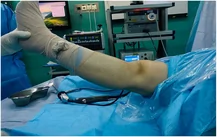

**VIDEO 1 atn270346-fig-1001:** Arthroscopic‐assisted combined anterior cruciate ligament and oblique popliteal ligament reconstruction of the left knee in a patient with symptomatic genu recurvatum. Video content can be viewed at https://doi.org/10.1002/atn2.70346.

As one of the 5 expansions of the semimembranosus muscle tendon, the oblique popliteal ligament (OPL) is a well‐defined, broad, flat fibrous band that forms a key part of the complex posterior knee anatomy.^1^, ^2^, ^3^ It projects superolaterally from the posteromedial joint capsule and tibial plateau to the posterolateral capsule at the medial margin of the lateral femoral condyle, forming an acute angle with the horizontal joint line.^2^, ^3^, ^4^ The OPL crosses the popliteal fossa from medial to lateral and serves as the primary static soft‐tissue restraint against knee hyperextension.^1^, ^3^ Its unique oblique orientation also enables the OPL to play a crucial role in stabilizing the knee by dividing the force vector into 2 horizontal and vertical limbs, particularly in resisting excessive external rotation forces.^4^, [Fig atn270346-fig-1001]

Despite being the largest ligament in the posterior knee, with significant biomechanical implications and vulnerability to involvement in posterior knee injuries,^3^, ^4^, ^5^ surgical strategies addressing posterior knee instability have historically focused on cruciate and posterolateral corner reconstructions. We propose the following recommended indications for OPL reconstruction, either isolated or combined with the anterior cruciate ligament (ACL), posterior cruciate ligament (PCL), or multiligament reconstructions, to help restore the native knee kinematics:

• Revision ACL reconstruction after recurrent graft failure, particularly in the presence of capsuloligamentous insufficiency^6^, ^7^
• Primary ACL reconstruction with clinically evident hyperextension tendency (genu recurvatum) on physical examination or abnormal tibial plateau slope^6^, ^8^, ^9^
• High‐grade pivot shift (≥ grade 2+) suggestive of rotational and posterior capsular laxity
• Symptomatic post‐traumatic nonosseous genu recurvatum (>5° compared with the contralateral limb) or detectable posterior capsular laxity^10^, ^11^
• Multiligament knee injuries, especially bicruciate reconstructions (ACL + PCL) requiring reinforcement of posterior stability
• Posterolateral instability with excessive external rotation on the dial test or persistent rotational laxity despite posterolateral corner reconstruction

We hereby describe an arthroscopic‐assisted surgical technique for OPL reconstruction, performed alone or in combination with ACL, PCL, posterolateral corner, or multiligament knee reconstructions. The goal is to restore posterior capsular tension, prevent symptomatic hyperextension, and reduce excessive external rotation. The surgical technique is demonstrated in Video 1.

## SURGICAL TECHNIQUE

### Physical Examination

Under spinal or general anesthesia and prior to skin incision, a standard knee physical examination is performed, with particular attention to passive knee extension, side‐to‐side hyperextension asymmetry, and posterior capsular end‐point quality. Heel‐height difference and degree of recurvatum are documented when feasible. Figure 1 shows a patient's knee with an ACL and concomitant medial meniscus injury exhibiting recurvatum during the preoperative physical examination. All subsequent surgical figures are from this patient's procedure (used with written informed consent).

**FIGURE 1:**
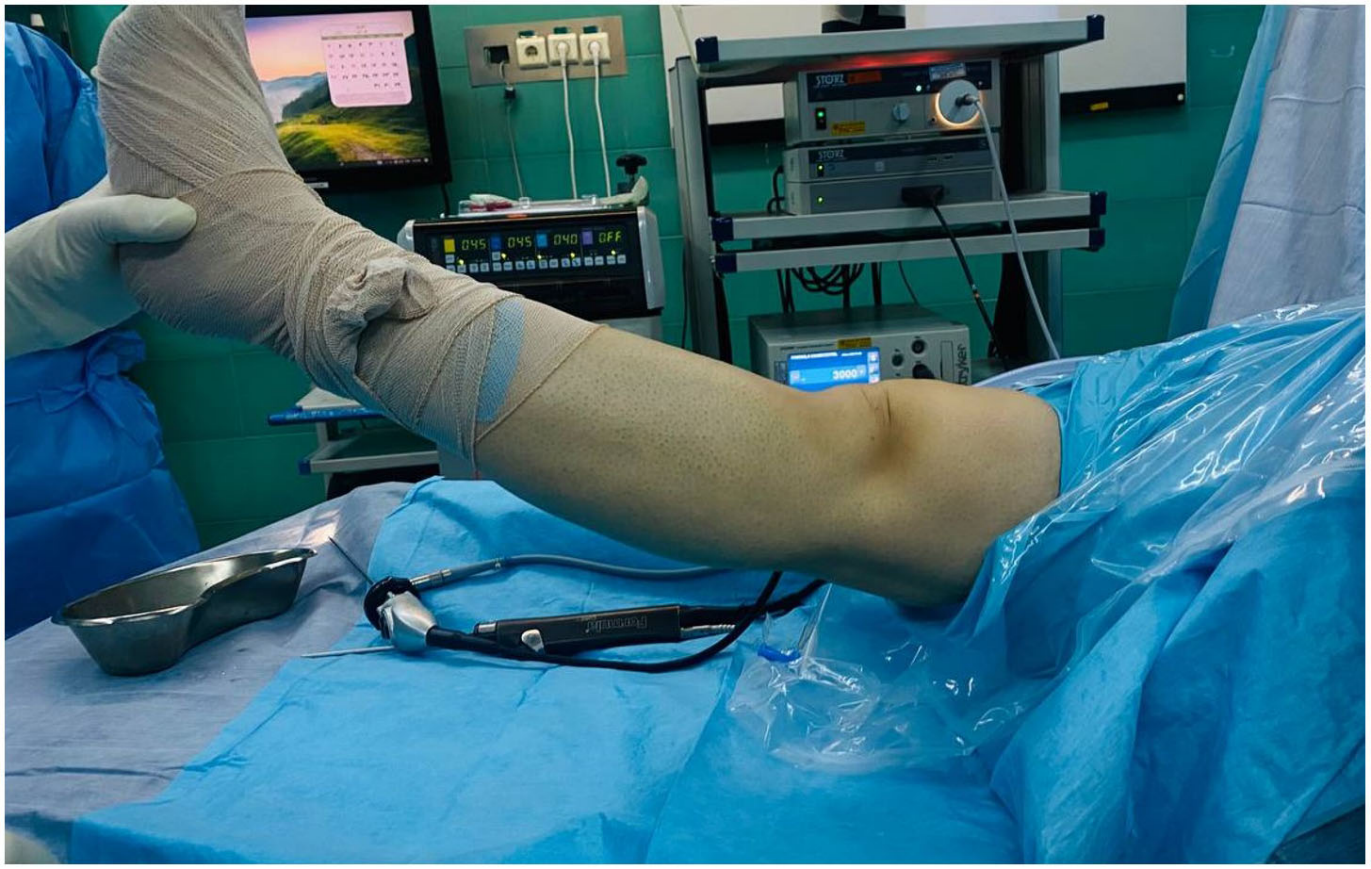
Patient position: The patient was positioned supine during surgery. Preoperative physical examination of the left knee in a supine patient with ACL tear and concomitant medial meniscus injury showing pathologic genu recurvatum. These findings suggest posterior capsular insufficiency and abnormal extension laxity, supporting the indication for OPL reconstruction. (ACL, anterior cruciate ligament; OPL, oblique popliteal ligament.)

### Arthroscopic Evaluation

First, the high‐thigh tourniquet is inflated, and a routine diagnostic knee arthroscopy is performed using the standard anterolateral and anteromedial portals. The cruciate ligaments, menisci, cartilage, and medial joint opening are assessed. Associated intra‐articular pathology is addressed according to standard practice. Figure 2 shows high‐grade ACL tearing with concurrent longitudinal tear of the medial meniscus in this patient.

**FIGURE 2:**
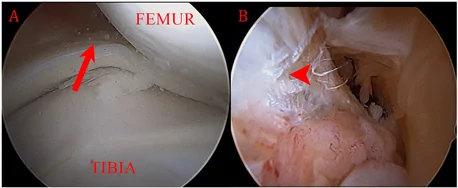
Patient position: The patient was positioned supine during surgery. Diagnostic arthroscopic evaluation of the left knee performed with the patient supine using the anterolateral viewing portal. (A) Arthroscopic visualization of a longitudinal tear of the medial meniscus within the medial compartment (red arrow). (B) Arthroscopic image showing a high‐grade ACL tear at its femoral attachment site with loss of normal fiber continuity (red arrowhead). These intra‐articular findings confirm associated structural pathology commonly encountered in knees with symptomatic instability and recurvatum. (ACL, anterior cruciate ligament.)

### Cruciate Tunnel Preparation

When OPL reconstruction is combined with cruciate ligament tears, first, the femoral and tibial tunnels for the cruciate of interest are prepared in a standard manner, regardless of the graft used (bone–patellar tendon–bone, hamstring autograft, quadriceps tendon autograft, or allograft) or the fixation method of choice (Figure 3). The cruciate graft is not passed through the tunnels in this stage of the operation, allowing unobstructed posterior work and minimizing the risk of tunnel convergence.

**FIGURE 3:**
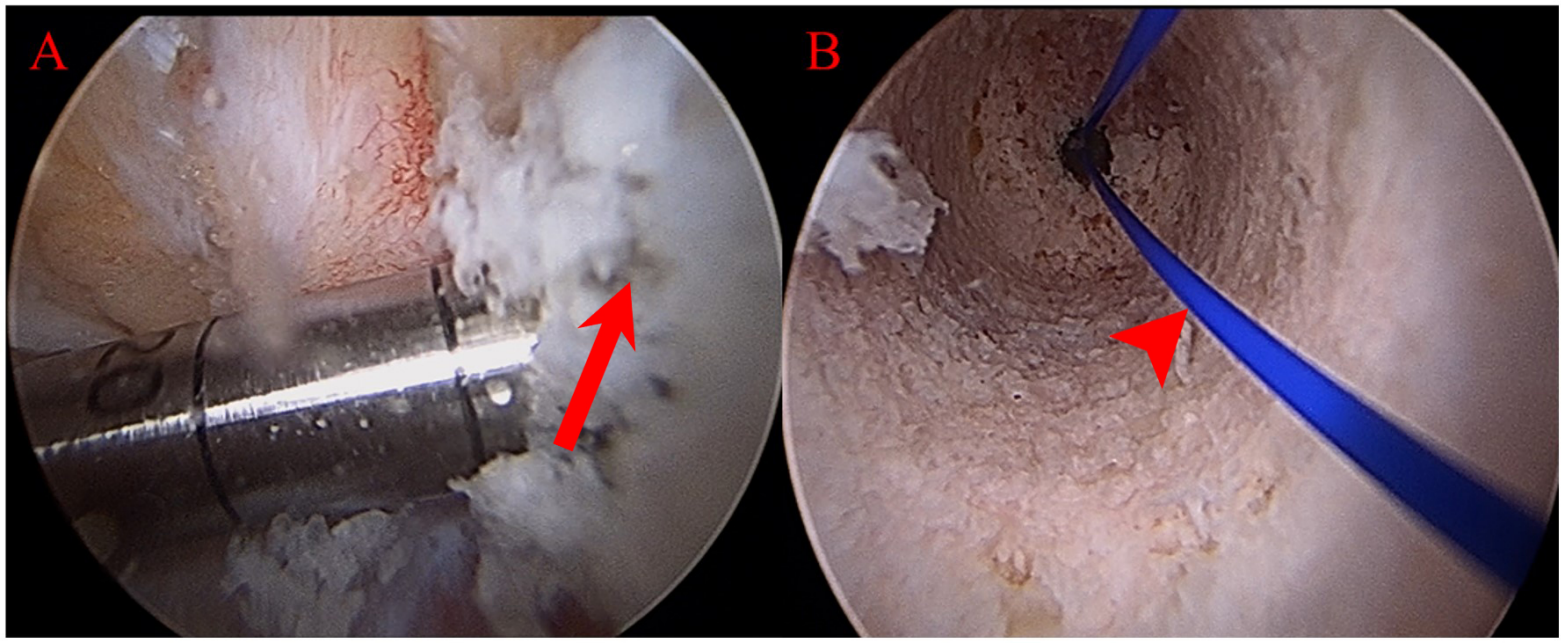
Patient position: The patient was positioned supine during surgery. Preparation of the ACL femoral tunnel during combined ACL and oblique popliteal ligament reconstruction of the left knee. (A) Arthroscopic view from the anterolateral portal showing femoral tunnel reaming using a standard ACL guide positioned at the native femoral ACL footprint (red arrow). (B) A PDS shuttle suture loop (red arrowhead) is passed through the femoral tunnel, and appropriate tunnel position and intratunnel orientation are confirmed through the anteromedial portal. This sequence shows standard cruciate tunnel preparation prior to posterior capsular reconstruction. (ACL, anterior cruciate ligament; PDS, polydioxanone.)

### Posterior Portal Creation

Standard posteromedial and posterolateral portals are established using hypodermic needles inserted into the joint space from outside‐in and confirmed by views from anterolateral and anteromedial portals. Then, a trans‐septal posterior portal is created using the method described by Ahn et al.^12^ to facilitate posterior graft passage (Figure 4). Because the popliteal neurovascular bundle lies anterior to the posterior capsule and OPL, all posterior work is performed with the knee flexed and under continuous arthroscopic visualization.^13^, ^14^ Blunt dissection and controlled instrument advancement are emphasized to minimize neurovascular risk.

**FIGURE 4:**
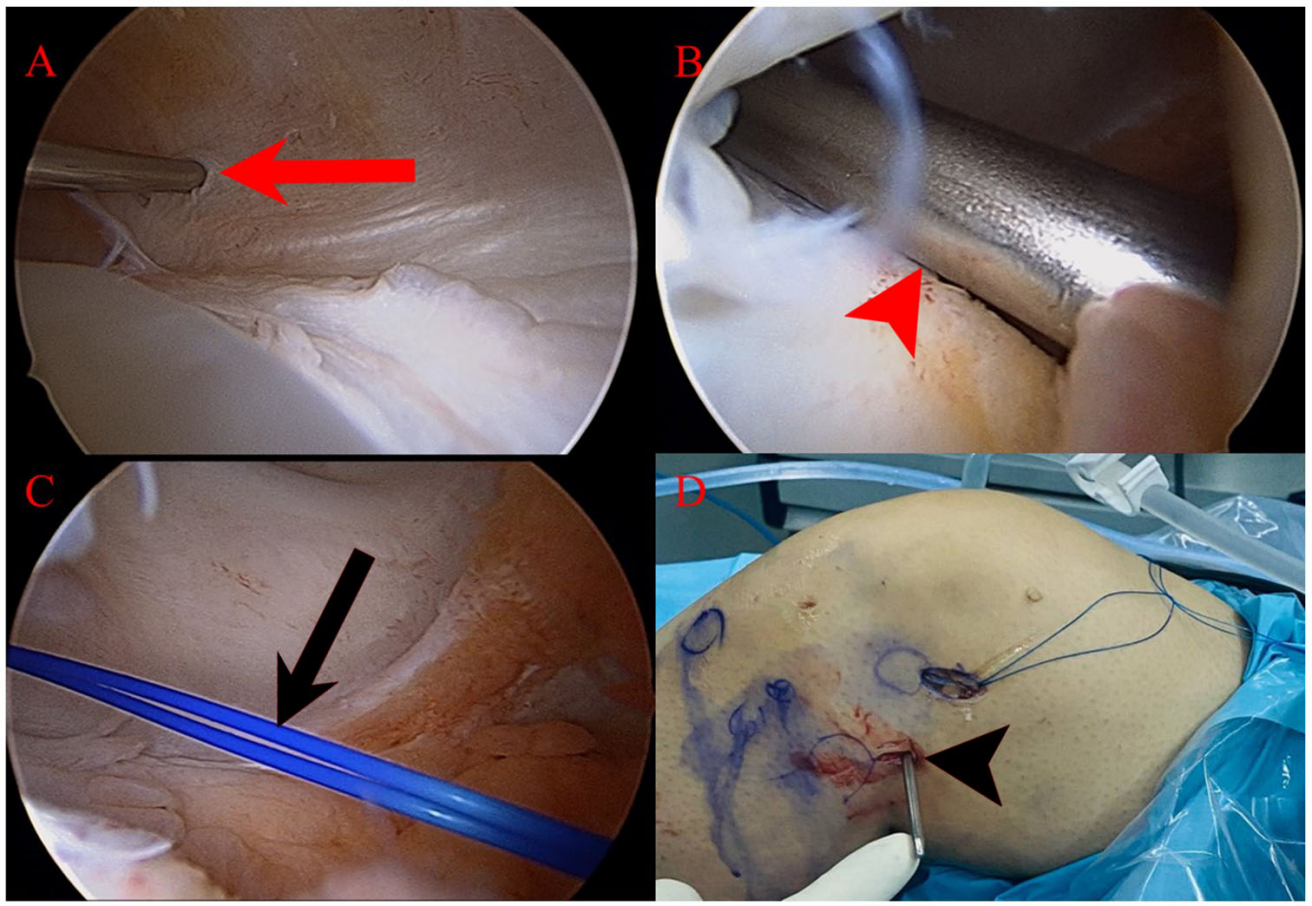
Patient position: The patient was positioned supine during surgery. Creation of posterior arthroscopic portals during OPL reconstruction of the left knee with the knee flexed to minimize neurovascular risk. (A) Establishment of the posteromedial portal under arthroscopic visualization from the anterolateral portal using an outside‐in spinal needle technique (red arrow). (B) Creation of the trans‐septal posterior portal using a blunt‐tipped mandrel pin passed across the posterior septum (red arrowhead). (C) Passage of a PDS shuttle suture from the posteromedial portal through the trans‐septal corridor and retrieval through the posterolateral portal (black arrow). (D) External view showing the location of the completed posterolateral portal (black arrowhead). These portals create a controlled posterior working corridor for safe graft passage across the posterior capsule. (OPL, oblique popliteal ligament; PDS, polydioxanone.)

### Femoral Tunnel for OPL

A 7‐mm‐diameter, 30‐mm‐deep femoral tunnel is reamed just proximal and posterior to the lateral epicondyle (approximating the native capsular attachment on the lateral femoral condyle) using a standard ACL guide pin under fluoroscopic guidance, ensuring a safe distance from any cruciate femoral tunnel (Figure 5).

**FIGURE 5:**
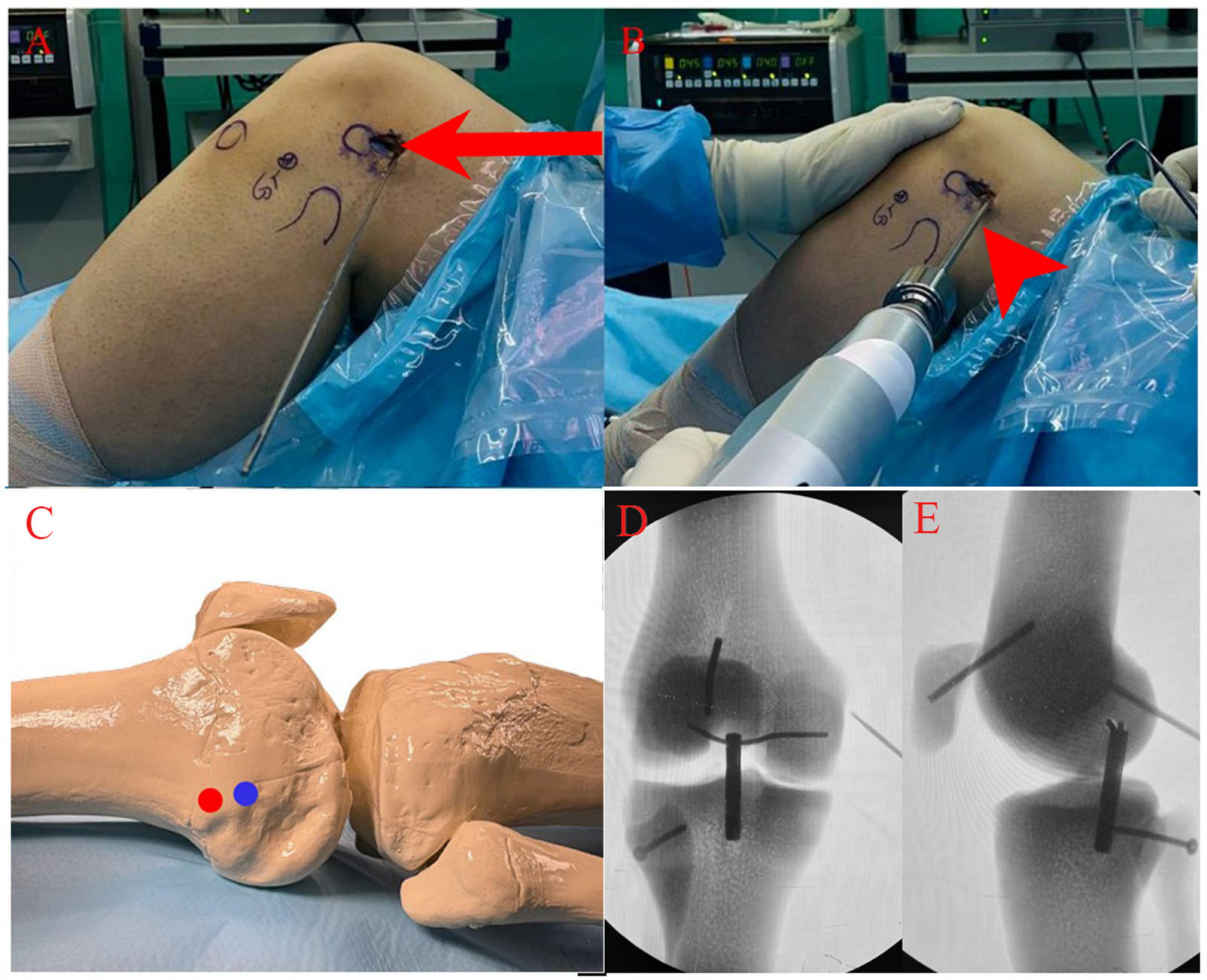
Femoral tunnel preparation for OPL reconstruction of the left knee. (A) A guide pin is inserted under fluoroscopic guidance just proximal and posterior to the lateral femoral epicondyle, approximating the native femoral attachment of the OPL (red arrow). (B) The femoral tunnel is reamed to a diameter of 7 mm and depth of 30 mm (red arrowhead), followed by passage of a PDS shuttle suture loop. (C) Sawbone model showing the relation between the OPL femoral tunnel (red dot) and the lateral femoral epicondyle (blue dot). (D,E) Anteroposterior and lateral fluoroscopic images of the sawbone model showing appropriate guide pin trajectory and tunnel positioning while avoiding convergence with cruciate reconstruction tunnels. This step establishes the femoral fixation site for recreation of the native oblique course of the OPL. (OPL, oblique popliteal ligament; PDS, polydioxanone.)

### Tibial Tunnel for OPL

A tibial guide pin is inserted at the posteromedial tibia, about 20 mm distal to the joint line along the posteromedial crest. After fluoroscopic confirmation, the tunnel is then reamed with a 7‐mm reamer to a depth of 30 mm. Care is taken to avoid interference between the cruciate and OPL tunnels (Figure 6).

**FIGURE 6:**
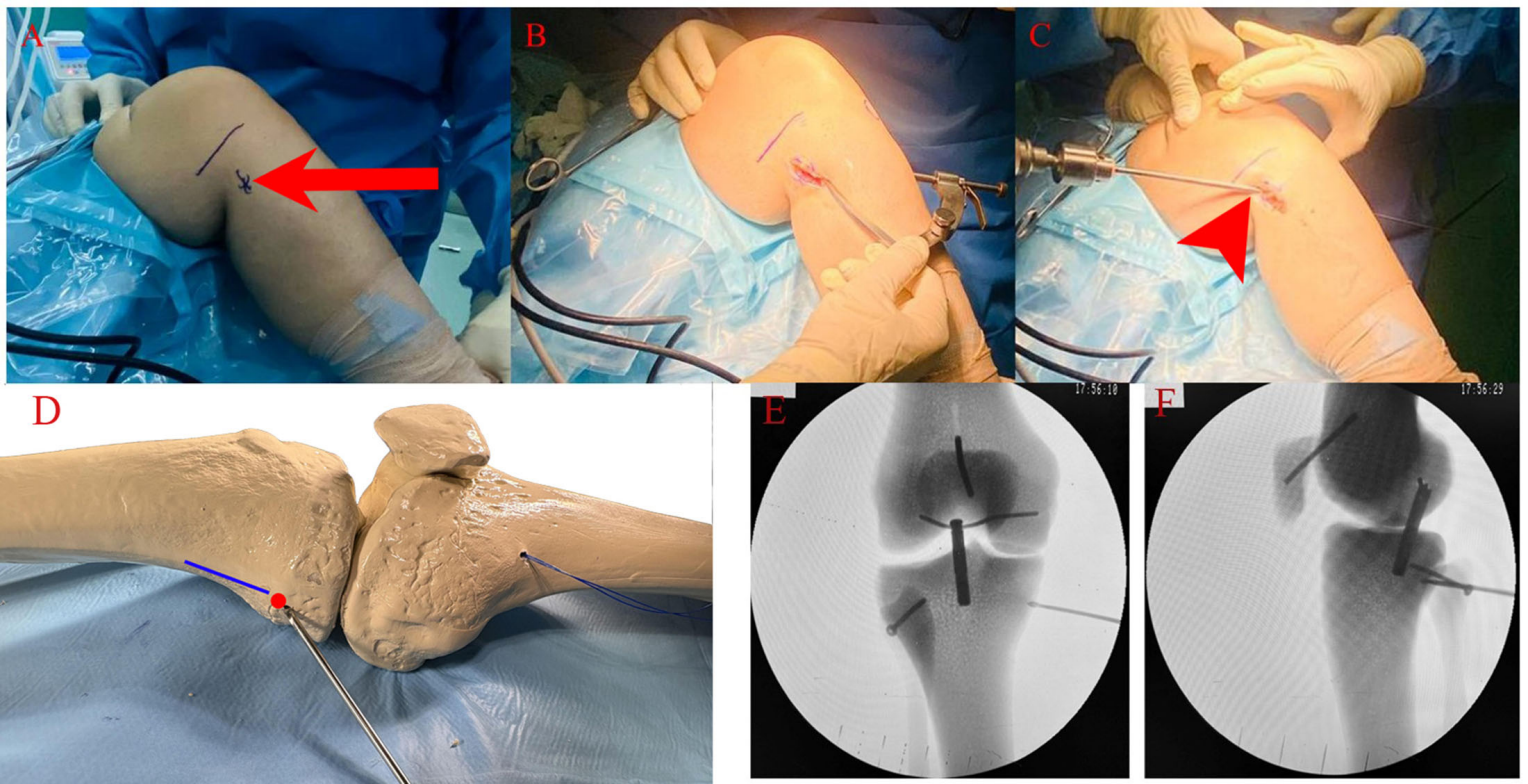
Tibial tunnel preparation for the OPL reconstruction of the left knee. (A) The intended tibial attachment site is identified approximately 2 cm distal to the joint line along the posteromedial tibial crest (red arrow). (B,C) Fluoroscopic guidance is used to confirm guide pin placement prior to reaming a 7‐mm‐diameter tunnel to a depth of 30 mm (red arrowhead). (D) Sawbone model showing the OPL tibial tunnel position (red dot) relative to the posteromedial tibial crest (blue line). (E,F) Fluoroscopic anteroposterior and lateral views of the sawbone model showing safe tibial tunnel orientation and avoidance of convergence with cruciate tunnels. This tunnel reproduces the posteromedial tibial attachment site of the native OPL. (OPL, oblique popliteal ligament.)

### Cruciate Graft Passage and Fixation

The cruciate graft is passed through the previously prepared femoral and tibial tunnels as per the desired reconstruction technique. An arthroscopic assessment of the reconstructed ACL (in this index case) is performed through the anterolateral portal (Figure 7).

**FIGURE 7:**
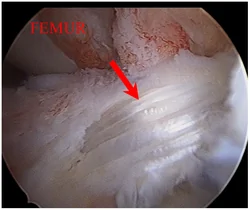
Patient position: The patient was positioned supine during surgery. Final fixation of the ACL graft during combined ACL and OPL reconstruction of the left knee. Arthroscopic evaluation through the anterolateral portal shows the reconstructed ACL graft (red arrow) positioned anatomically within the intercondylar notch after passage through the prepared femoral and tibial tunnels. Femoral fixation is achieved using either suspensory fixation or an interference screw technique. This step restores primary anterior stability prior to posterior capsular reconstruction. (ACL, anterior cruciate ligament; OPL, oblique popliteal ligament.)

### OPL Graft Passage

A tibialis posterior or hemi‐Achilles allograft, trimmed to a diameter of approximately 7 mm (or folded as needed to fit the 7‐mm tunnels), is preferred by the authors for OPL reconstruction. Although the native OPL is a broad, flat structure, a cylindrical graft is selected to enhance reproducibility and fixation reliability. The OPL graft loop is passed through the tibial tunnel and then shuttled across the posterior capsule via the posteromedial to trans‐septal to posterolateral route. Then, the free end of the graft is delivered into the femoral tunnel (Figure 8). The isometry of the graft is checked by holding manual tension on the graft ends and cycling the knee through the full range of motion.

**FIGURE 8:**
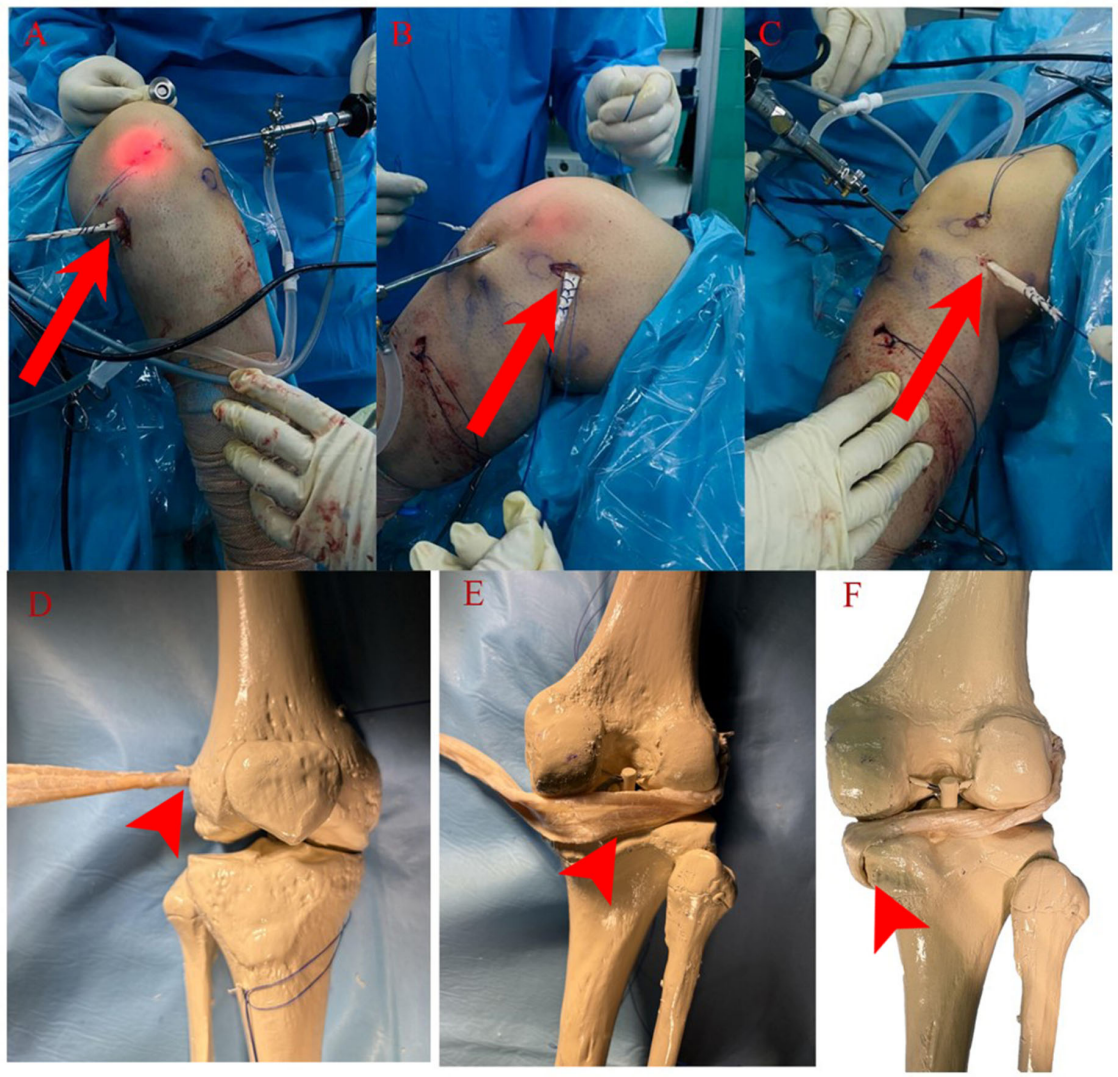
Arthroscopic‐assisted graft passage during OPL reconstruction of the left knee. (A‐C) Sequential intraoperative photographs obtained through the posterior portals showing passage of the OPL graft from the tibial tunnel into the posterior compartment (red arrows). The graft is advanced anterior to the posterior capsule and posterior to the PCL, recreating the native oblique trajectory of the OPL from the posteromedial tibia to the posterolateral femur. (D‐F) Corresponding sawbone model images showing the final graft orientation and posterior capsular course (red arrowheads). This technique enables controlled graft passage while minimizing risk to the popliteal neurovascular structures. (OPL, oblique popliteal ligament; PCL, posterior cruciate ligament.)

### Femoral and Tibial Tunnel Fixation

First, the OPL graft is fixed at the femoral side. Fixation is achieved with a 7 × 30 mm interference screw (LINVATEC, CONMED, Largo, FL). Then, the tibial side is fixed similarly using a 7 × 30 mm interference screw (LINVATEC, CONMED), applying approximately 20 N of manual tension at 20° of knee flexion (Figure 9). This slightly flexed position of the knee during graft fixation helps the graft to reach maximum tension in full extension to prevent genu recurvatum. Final arthroscopic views confirm appropriate tension (lax in flexion, taut in extension) and anatomic positioning (Figure 10).

**FIGURE 9:**
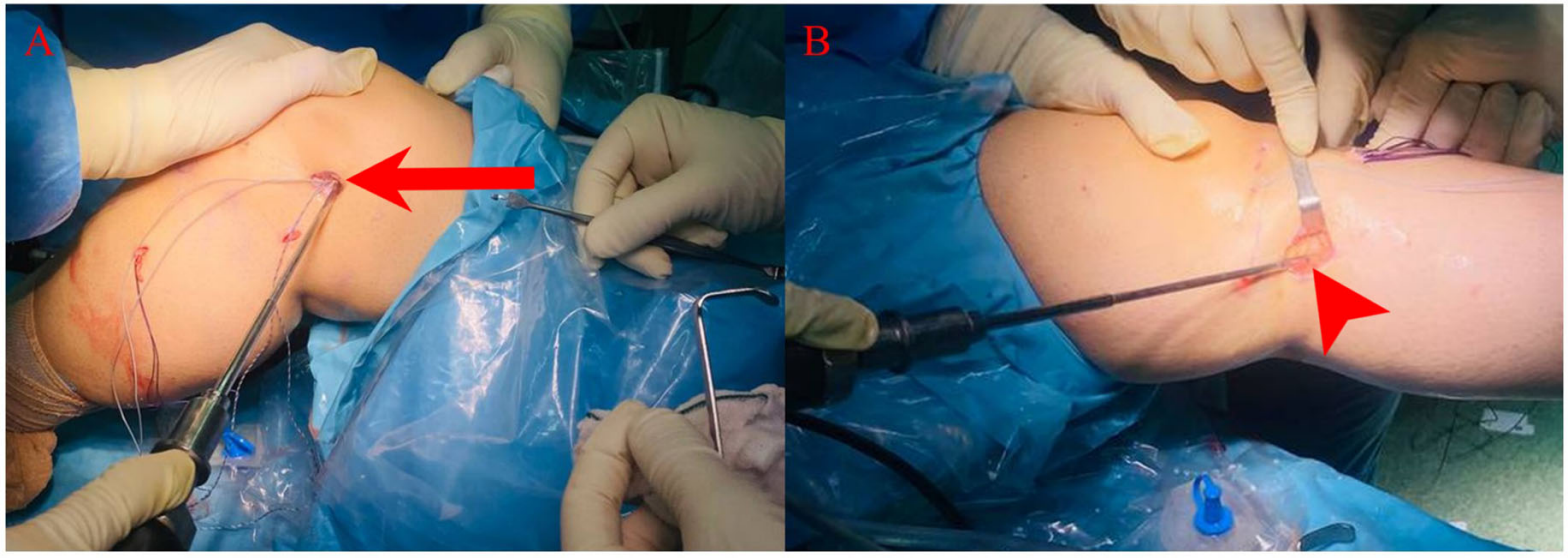
Patient position: The patient was positioned supine during surgery. Fixation of the OPL graft in the left knee. (A) Femoral fixation is performed first using a 7 × 30 mm interference screw (LINVATEC, CONMED, Largo, FL) inserted into the femoral tunnel (red arrow). (B) Tibial fixation is subsequently completed using a similar interference screw (red arrowhead) while applying manual graft tension with the knee maintained at approximately 20° of flexion to optimize posterior capsular tension in full extension. This fixation strategy aims to restore resistance to genu recurvatum while preserving physiologic flexion laxity. (OPL, oblique popliteal ligament.)

**FIGURE 10:**
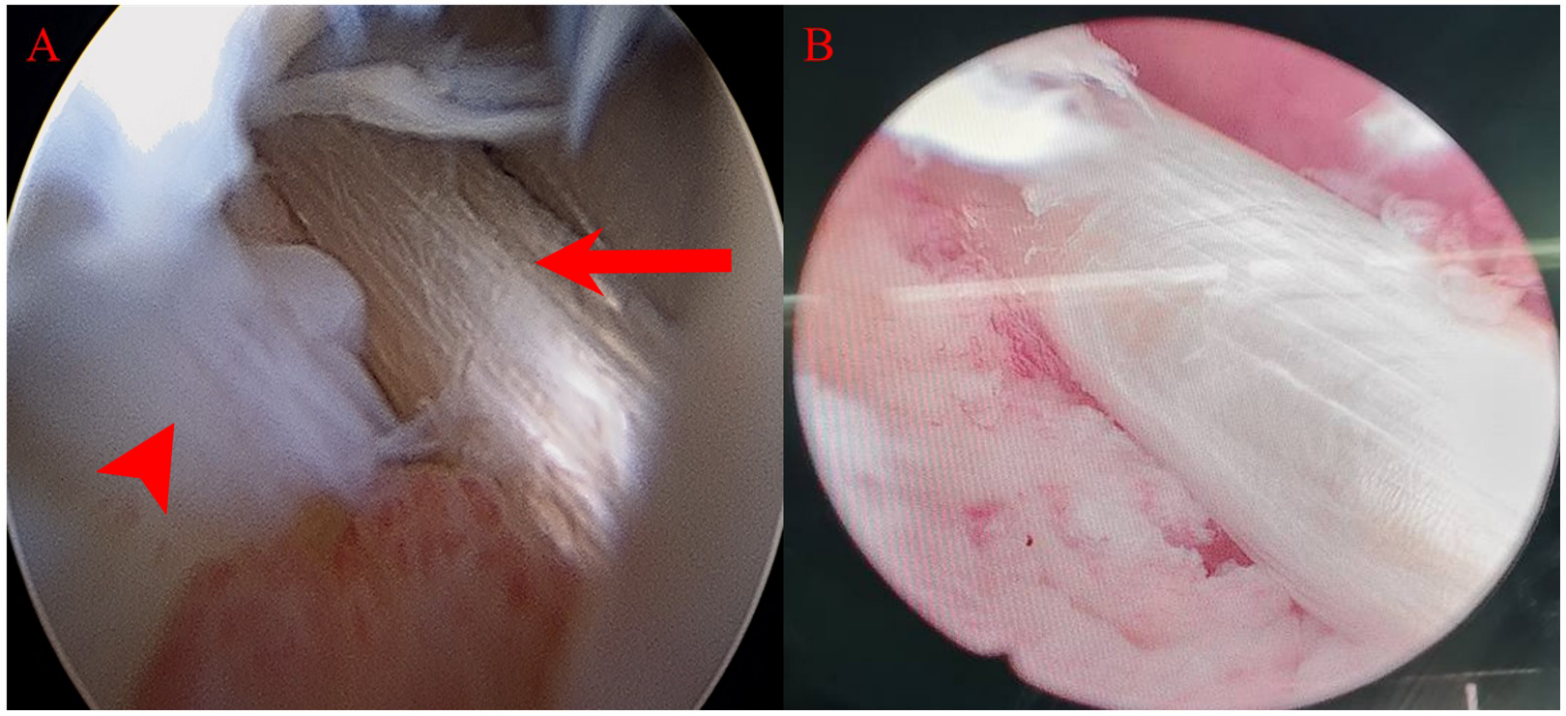
Patient position: The patient was positioned supine during surgery. Final arthroscopic assessment of the reconstructed OPL graft of the left knee. (A) Arthroscopic visualization through the posterior compartment shows the reconstructed OPL graft (red arrow) positioned posterior to the posterior cruciate ligament (red arrowhead). (B) Final assessment confirms appropriate graft tension, anatomic orientation, and restoration of posterior capsular stability, with the graft remaining taut in extension and lax in flexion. These findings verify successful recreation of the native posterior stabilizing anatomy. (OPL, oblique popliteal ligament.)

### Postoperative Rehabilitation

The postoperative rehabilitation protocol for OPL reconstruction varies based on institutional preferences for standard cruciate or multiligament reconstructions, with no specific restrictions imposed by the OPL procedure. Forceful hyperextension is avoided early in recovery (Figure 11). Extension bracing may be considered in patients with significant preoperative recurvatum. Return‐to‐sport timelines are dictated by concomitant procedures. Tables 1 and 2 summarize the “advantages and disadvantages” and “pearls and pitfalls” of the presented surgical technique.

**FIGURE 11:**
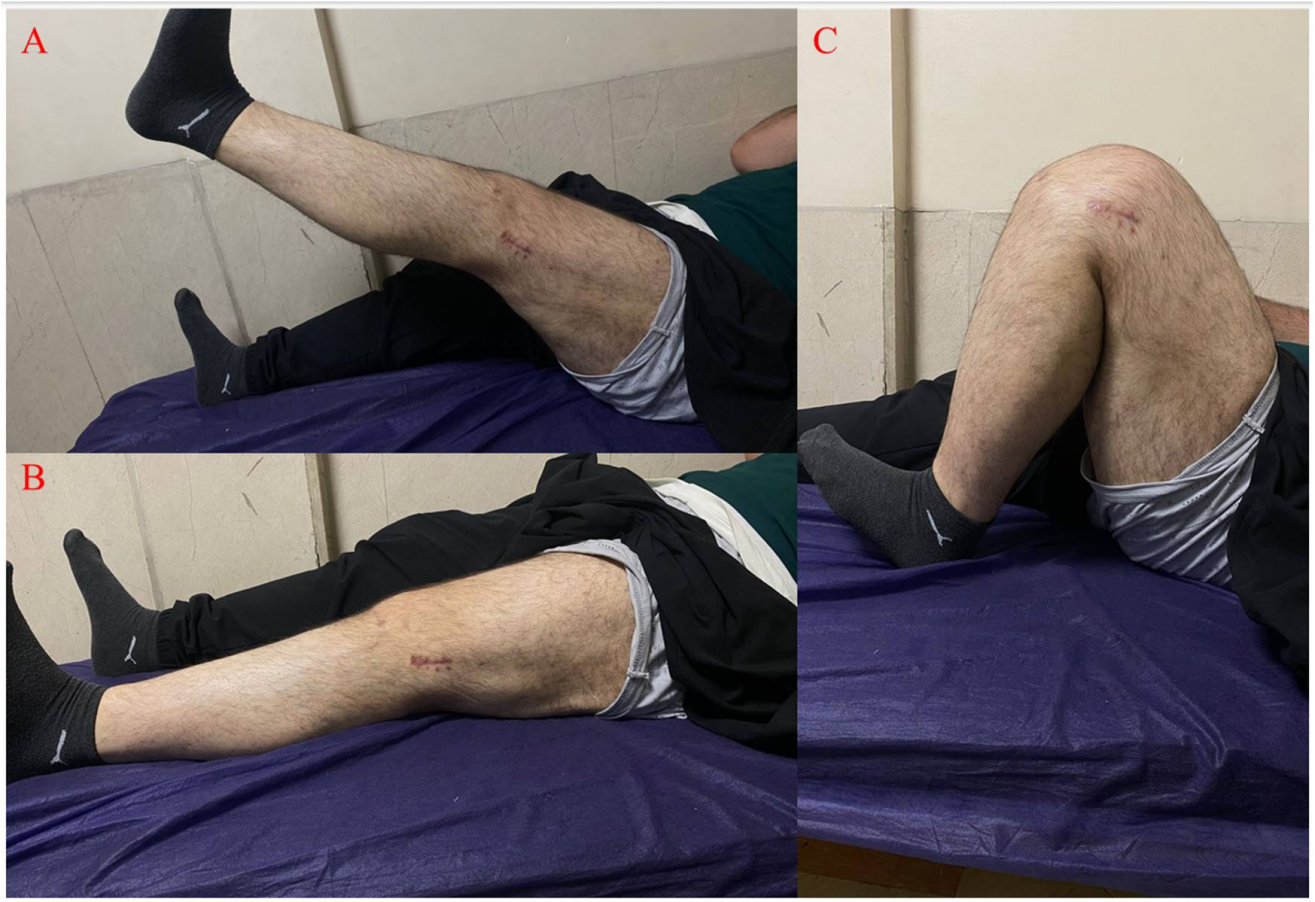
Patient position: The patient was positioned supine during surgery. Clinical examination at 2 months after combined ACL and OPL reconstruction of the left knee. (A) Straight‐leg raise showing active quadriceps control without extensor lag. (B) Full knee extension without residual genu recurvatum or hyperextension deformity. (C) Full knee flexion achieved painlessly during postoperative rehabilitation. These images show early restoration of motion and functional stability after posterior capsular reconstruction. (ACL, anterior cruciate ligament; OPL, oblique popliteal ligament.)

**TABLE 1:** Advantages and Disadvantages of Arthroscopic OPL Reconstruction

**Advantages**	**Disadvantages**
Anatomically targets posterior capsular insufficiency and reconstructs the OPL directly	Technically demanding procedure requiring advanced posterior knee arthroscopy skills
May restore native restraint against genu recurvatum and excessive hyperextension	Proximity to the popliteal neurovascular bundle increases risk of iatrogenic injury
Potential adjunctive control of rotational instability and excessive external tibial rotation	Lack of long‐term clinical outcome data
Can be combined with ACL, PCL, PLC, or multiligament reconstructions in a single‐stage procedure	Potential tunnel convergence with concomitant cruciate reconstructions
Arthroscopic‐assisted approach minimizes soft‐tissue dissection compared with open posterior procedures	Native OPL anatomy is broad and flat, whereas reconstruction uses a cylindrical graft
Allows standardized tunnel placement and reproducible graft passage	Indications and patient selection criteria are not yet fully established
Posterior graft passage through trans‐septal portals allows controlled visualization during reconstruction	Requires fluoroscopic guidance and familiarity with posterior portal creation
May be particularly beneficial in patients with recurrent ACL failure associated with hyperextension	Additional operative time and procedural complexity in combined ligament surgery
Tensioning in slight flexion may optimize posterior capsular restraint in extension	Potential risk of postoperative overconstraint or loss of extension if improperly tensioned
Compatible with multiple graft types and fixation methods used in ligament reconstruction	Rehabilitation protocols remain nonstandardized, specifically for OPL reconstruction

ACL, anterior cruciate ligament; OPL, oblique popliteal ligament; PCL, posterior cruciate ligament; PLC, posterolateral corner.

**TABLE 2:** Pearls and Pitfalls of Arthroscopic OPL Reconstruction

Pearls	Pitfalls
Perform careful preoperative assessment of recurvatum, heel‐height difference, and posterior capsular laxity	Failure to recognize posterior capsular insufficiency may lead to persistent instability
Prepare cruciate tunnels before posterior work to improve visualization and reduce tunnel conflict	Early graft passage before tunnel preparation may obstruct posterior compartment access
Maintain the knee in flexion during posterior arthroscopy to increase the safety distance from neurovascular structures	Posterior instrumentation in extension increases neurovascular risk
Use continuous arthroscopic visualization during posterior graft passage	Blind or forceful graft passage may injure posterior structures
Establish posteromedial, trans‐septal, and posterolateral portals carefully using blunt dissection techniques	Aggressive septal penetration may damage the popliteal vessels
Ensure adequate separation between the cruciate and OPL femoral tunnels	Tunnel convergence may compromise fixation strength
Confirm graft isometry by cycling the knee through full range of motion before fixation	Failure to assess isometry may result in graft overconstraint or laxity
Fix the graft with the knee at approximately 20° flexion to optimize tension in full extension	Fixation in excessive flexion or full extension may lead to abnormal graft tensioning
Verify final graft behavior arthroscopically: taut in extension and lax in flexion	Persistent graft laxity in extension may fail to control recurvatum
Use fluoroscopic guidance for accurate tunnel positioning	Malpositioned tunnels may alter graft biomechanics and compromise function
Avoid forceful postoperative hyperextension during early rehabilitation	Premature hyperextension loading may stretch the reconstruction
Consider extension bracing in patients with severe preoperative recurvatum	Inadequate postoperative protection may predispose to recurrent hyperextension instability

OPL, oblique popliteal ligament.

## DISCUSSION

As the largest ligament of the posterior knee complex, the OPL plays a crucial role in knee joint static and dynamic stability, considering the specific course of the ligament.^3^, ^4^ This technical note describes a dedicated attempt to anatomically reconstruct the OPL, addressing a gap in the surgical management of posterior capsular insufficiency. Although the OPL has received relatively limited attention in the literature compared with other major stabilizers like the cruciate ligaments, its pivotal role as a static restraint to knee hyperextension is now well‐established.^3^, ^15^

Derived from the tendon of the semimembranosus muscle, the OPL runs obliquely upward and laterally across the popliteal fossa.^1^, ^2^, ^3^ It functions as the primary ligamentous restraint to knee hyperextension.^3^ Biomechanical studies using sequential sectioning revealed that the greatest increase in knee hyperextension occurred after sectioning the OPL, a finding that was independent of the cutting order. This resultant laxity often approached or exceeded the increases observed after sectioning the ACL and PCL combined.^3^ Additionally, the OPL strengthens the posterior central region of the knee joint capsule and aids in rotatory stability through its tibial attachment, promoting overall joint integrity.^2^, ^15^

For patients presenting with symptomatic post‐traumatic genu recurvatum, which impairs gait, particularly on uneven terrain, restoring OPL integrity is essential.^1^ Knees with a decreased posterior tibial slope exhibit more genu recurvatum after traumatic injury to static extension restraints and have more symptoms. Preoperative slope assessment helps identify candidates likely to benefit from OPL reconstruction.^3^ Existing approaches, such as posterior capsular plication or tibial slope‐modifying osteotomy, may indirectly address hyperextension but do not anatomically reconstruct the OPL itself.^7^, ^10^, ^11^

The role of the OPL as the primary restraint against genu recurvatum is well‐established. We hypothesize, however, that its distinctive oblique course from the posteromedial tibia to the posterolateral femur, which in many ways mirrors the ACL's orientation but in the posterior plane, may also contribute to resisting high‐grade pivot shift by enhancing posterior capsular tension and limiting anterolateral rotatory subluxation, as well as providing subtle restraint against valgus laxity in extension. Additionally, this medial‐to‐lateral cross‐knee attachment likely helps control excessive external tibial rotation (Figure 12). Although these secondary stabilizing effects appear plausible based on the ligament's anatomy, they remain speculative and await confirmation through dedicated biomechanical studies.

**FIGURE 12:**
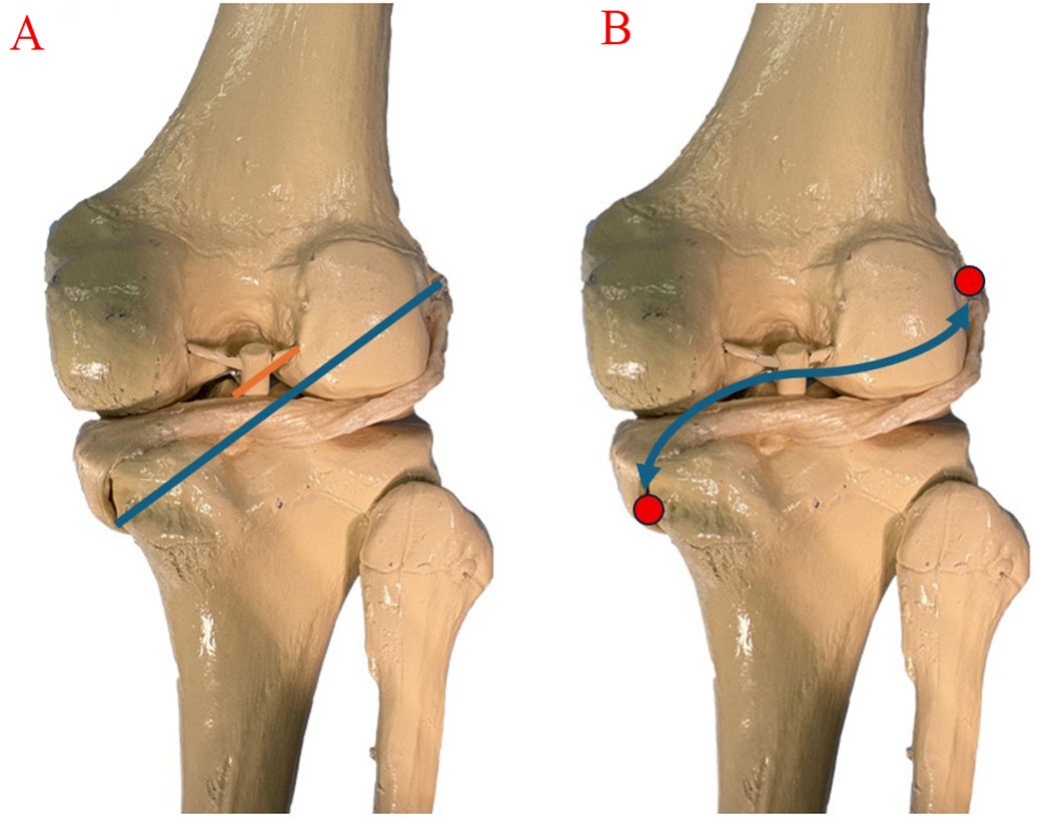
Proposed biomechanical role of the OPL in knee stability. (A) Schematic illustration showing the oblique orientation of the OPL (blue line) extending from the posteromedial tibia to the posterolateral femur relative to the ACL (orange line). This configuration may contribute to resistance against hyperextension, pivot shift, and valgus instability. (B) Illustration showing how the medial‐to‐lateral orientation of the OPL may help resist excessive external tibial rotation by reinforcing posterior capsular tension. These proposed secondary stabilizing roles remain theoretical and require further biomechanical validation. (ACL, anterior cruciate ligament; OPL, oblique popliteal ligament.)

The dimensions of the native OPL vary significantly between studies and populations, underscoring the need for standardized measurement protocols. For example, mean length measurements have been reported between 28.9 and 45.6 mm. Furthermore, width measurements (e.g., 17.5 mm at the origin) are often significantly larger than thickness measurements (e.g., 7.3 mm), contributing to inconsistencies in the literature.^1^, ^15^ Although aware of these variations, for the sake of simplicity and reproducibility, our technique aims to create standardized tunnels (7 mm diameter, 30 mm depth) to restore tension across the posterior capsule, recognizing that the reconstructed graft must function within this wide range of native anatomical configurations.

Reconstruction of the OPL is inherently challenging because of its location in the popliteal fossa, which contains vital neurovascular structures. Surgeons must maintain a detailed knowledge of this posterior anatomy to avoid iatrogenic injury. The popliteal vein and artery lie superficial (anteriorly) to the OPL, and critical structures like the genicular neurovascular structures pierce the OPL itself. The use of controlled graft passage under arthroscopic visualization via the posteromedial, trans‐septal, and posterolateral routes in knee flexion, as described, is crucial for mitigating this risk. Key technical considerations include ensuring the femoral OPL tunnel is sufficiently distant from the cruciate femoral tunnel to prevent convergence and tensioning the OPL graft in slight knee flexion prior to fixation to achieve proper posterior capsular tension in full extension.

Future research must focus on biomechanical studies and long‐term clinical series to validate this technique. Additional studies are required to determine if surgical reconstruction can successfully restore normal motion limits in patients with symptomatic genu recurvatum. A better understanding of OPL pathology may lead to improved management of such related conditions.

## DISCLOSURES

The authors (F.A.O., M.M.S., A.M.R., M.M.O., A.A., M.D., A.P.) declare that they have no known competing financial interests or personal relationships that could have appeared to influence the work reported in this paper.
